# Promising strategies in natural products treatments of psoriasis-update

**DOI:** 10.3389/fmed.2024.1386783

**Published:** 2024-09-04

**Authors:** Sihua Le, Xuan Wu, Yuan Dou, Tianhao Song, Hongyang Fu, Hongbin Luo, Fan Zhang, Yi Cao

**Affiliations:** ^1^Ningbo Medical Center LiHuiLi Hosptial, Ningbo, China; ^2^The First School of Clinical Medicine, Zhejiang Chinese Medical University, Hangzhou, China; ^3^The First Affiliated Hospital of Zhejiang Chinese Medical University (Zhejiang Provincial Hospital of Traditional Chinese Medicine), Hangzhou, China

**Keywords:** psoriasis, natural product, pathogenesis, TCM, dermatology

## Abstract

Psoriasis is a chronic, relapsing, inflammatory skin disease and has been increasing year by year. It is linked to other serious illnesses, such as psoriatic arthritis, cardiometabolic syndrome, and depression, resulting in a notable decrease in the quality of life for patients. Existing therapies merely alleviate symptoms, rather than providing a cure. An in-depth under-standing of the pathogenesis of psoriasis is helpful to discover new therapeutic targets and develop effective novel therapeutic agents, so it has important clinical significance. This article reviews the new progress in the study of pathogenesis and natural products of psoriasis in recent years. These natural products were summarized, mainly classified as terpenoids, polyphenols and alkaloids. However, the translation of experimental results to the clinic takes a long way to go.

## 1 Introduction

Psoriasis is an immune-mediated chronic, inflammatory, and systemic illness that affects roughly 2 to 3% of the world’s population and has been increasing year by year (1). Its clinical manifestations are diverse, with typical symptoms of scaly erythema or plaques that are localized or widely distributed and are divided into vulgaris, arthropathy, herpes, and erythroderma types according to clinical features. In the real world, psoriasis can be told apart from other skin conditions by its red plaques with silver or white multilayered scales and a thicker acanthotic epidermis. Itching, aching sensations, and bleeding are common symptoms reported by patients (2). Psoriasis can appear anywhere but is most common on the dorsum of the elbows and knees, the extensor sides of the extremities, in the sacral region, and the scalp. It is symmetrical on both sides. Frequently, psoriasis also affects the fingernail and toenail regions. Psoriasis lesions exhibit epidermal acanthosis, rete ridges, immune-cell infiltration in the dermis, and enhanced angiogenesis when examined histopathologically (3). As of now, the pathogenesis of psoriasis is complex and not entirely understood. Environmental, genetic, and immunological variables all play a role in pathogenesis, particularly innate and adaptive immunity. Several immune cells and cytokines are tightly linked to the pathophysiological process of psoriasis. Furthermore, psoriasis always reduces quality of life, and patients face social stigma and discrimination (4, 5).

Epidermal hyperplasia brought on by excessive keratinocyte (KC) proliferation is primarily what causes the pathogenic aspects of psoriasis. The pathophysiological process of psoriasis is tightly linked to several immune cells and cytokines. Topical therapy, physical therapy, and systemic therapy are all used to treat psoriasis. In patients with minor symptoms, external treatment was the main approach. For patients with moderate and severe symptoms, a combination of topical treatment with either physical therapy, traditional systemic medication therapy, or biological agents alone was employed (6). Furthermore, it is important to acknowledge that many of these approaches have the capacity to cause various negative outcomes in patients, such as atrophy, organ toxicity, immunosuppression, infection, and carcinogenesis. These side effects greatly restrict the long-term feasibility and practicality of using such medicines.

Hence, how to develop a new and effective therapy for psoriasis remains an urgent conundrum. Traditional medicine has been known and used in psoriasis for years, resulting in experience with therapies and prescriptions that provide the basis for the treatment of psoriasis. With deep research into traditional medicine through modern pharmacological and chemical means, many natural products with anti-psoriasis activity have been unlocked (7). We used the Pubmed database, with “natural products” and “psoriasis” as keywords, and the time limit was between 2000 and 2023.

**Figure 1:**
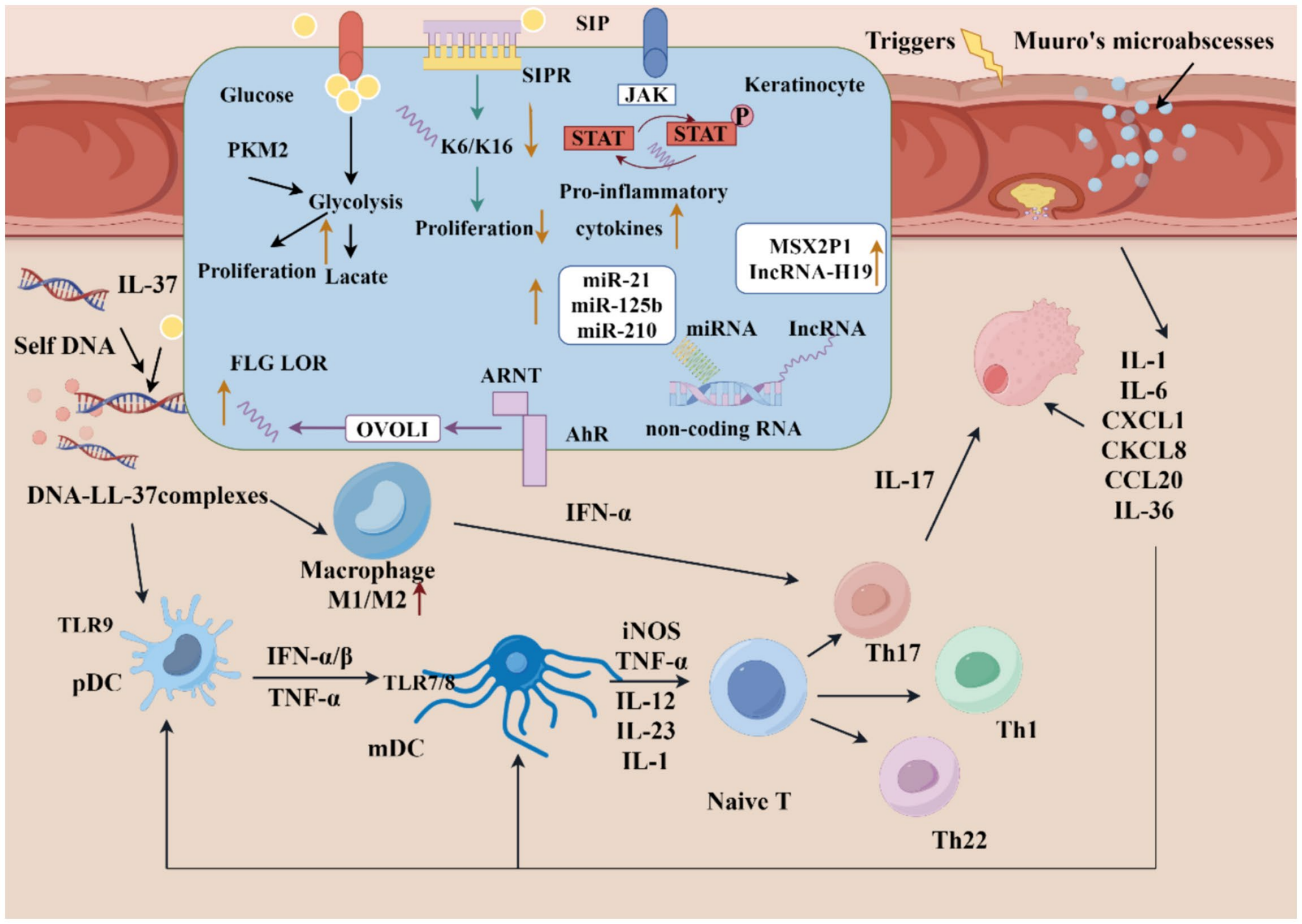
Pathologic mechanisms of psoriasis. Keratinocytes can be stimulated by initial triggers, then stressed keratinocytes release self-nucleotides, which combined with antimicrobial peptide (LL-37), activate pDCs and subsequent mDCs, involving in the initiation phase of psoriasis. In the development phase, adaptive immune responses are activated. The cytokines in the psoriatic lesions activate Th1, Th2, and Th17 cells for production of various cytokines to contribute to amplify the inflammation. The signaling of metabolic, S1P-S1PR, JAK–STAT, AhR pathway and epigenetic are dysregulated in keratinocyte. (1) Excessive proliferation of KCs can increase glucose uptake, then significantly level glycolysis up; (2) S1P can combined with different S1PR, then regulate KC differentiation and proliferation; (3) Active JAK-STAT signaling will promote proinflammatory cytokines transcription; (4) AhR pathway can upregulate the expression of FLG and LOR though OVO-1; (5) Dysregulated noncoding RNA of KCs (The figure made by figdraw).

Here, this article reviews the new progress in the pathogenesis of psoriasis (Figure 1) and natural products in recent years.

## 2 Pathologic mechanisms of psoriasis

The epidermis, dermis, and subcutaneous tissue comprise the skin, which serves as the body’s initial barrier against the ingress of foreign organisms. Maintaining the balance of skin function is what KCs do by interacting with bioactive substances like immune cells, cytokines, and antimicrobial peptides. The epidermis is mainly composed of KCs. Under typical physiological circumstances, the basal layer cells in the epidermis undergo continuous division, eventually differentiating and maturing into KCs. 28 days is the replacement interval for standard KCs. Conversely, psoriasis patients exhibit an accelerated proliferation of KCs, resulting in a reduction of their replacement time to approximately 3–4 days. So, when epidermal cells do not mature properly, it causes problems with keratinization and epidermal tissue proliferation, which can cause several harmful effects (8).

Researchers are still exploring the pathogenesis of psoriasis. Early studies mainly focused on the growth disorder of KCs. As scientific investigation has progressed, it has come to light that irregular KC proliferation may be the result of innate and adaptive immune disorders. The theory of the ‘IL-23/T helper 17 (IL-23/Th17) pathway’ in the pathogenesis of psoriasis, which Kastelein et al. and Fitch et al. identified and proposed in 2007, remains the foundational theory in psoriasis pathogenesis research and a significant target for pharmaceutical development (6, 9, 10). Predisposing factors for psoriasis include systemic factors (infection), local factors (skin injury, etc.), pharmaceutical substances, and psychological stress. KCs release a lot of antimicrobial peptides, such as catholicizing (LL-37), β-defensins, and S100 protein, in the early stages of psoriasis. This is because predisposing factors make them more active (11). LL-37 can form a complex with the genetic material of damaged cells. This complex turns on the TLR9 receptor in plasmacytoid dendritic cells (pDCs), which then causes a lot of interferon-α (IFN-α) and IFN-β to be released into the body. Not only do these interferons control how T cells grow and mature, but they also play a key role in the development of psoriasis by activating and maturing myeloid dendritic cells (mDCs) and triggering further effects (12). Subsequently, activated myeloid-derived suppressor cells (mDCs) release nitric oxide and nitric oxide synthase, which generates nitric oxide. This nitric oxide induces epidermal hyperplasia, inflammation, and the differentiation of helper T cells into various subsets of helper T cells (13). These T cell subsets migrated toward psoriatic lesions, where activated Th1 cells secreted IFN- and TNF- and Th17 cells secreted IL-17, which in turn stimulated impaired KC proliferation and differentiation (13). Then, KCs release more antimicrobial peptides and chemokines, which bring in more immune cells and make psoriatic disease worse (13).

Currently, targeted therapy with multiple cytokines has shown good efficacy in psoriasis, including TNF-α, IL-23, and IL-17 monoclonal antibodies. A variety of immune cells (T cells, DCs, and KCs) can release TNF-α, which plays multiple roles in the pathogenesis of psoriasis. These cells have pro-inflammatory activity and can interact with other cytokines to amplify inflammation. In addition, they work as cytokines ahead of the IL-23/Th17 axis and can make DCs make IL-23 (12). IL-23 has a close relationship with IL-17, as IL-17 promotes the proliferation and differentiation of Th17 cells. Also, IL-17 makes KCs more pro-inflammatory and makes them make the pro-inflammatory chemokine CCL20. This chemokine attracts Th17 and mDCs, which causes them to gather (12).

## 3 Pathogenesis of psoriasis

### 3.1 Il-36

Recent studies have found that the cytokine IL-36 plays a more important role in pustular psoriasis than other types of psoriasis. There are several IL-36 molecules, such as IL-36α, IL-36β, IL-36γ, and the IL-36 receptor antagonist (IL-36RN). They can all bind to the IL-36 receptor (IL-36R), but IL-36RN naturally blocks IL-36α, IL-36β, and IL-36γ (12). In psoriasis patients, the expression level of IL-36 is significantly increased in the skin lesions and serum and is positively correlated with the severity of the disease, and mutations or deletions in IL36RN, the gene encoding IL-36RN, can lead to the development of pustular psoriasis (14, 15).IL-36 is mainly expressed in epithelial cells and immune cells, and IL-17, TNF-α and IL-36 can induce their increased expression by themselves, which can activate NF-κB and MAPKs signaling pathways after binding to its receptor IL-36R and promote the expression and secretion of cytokines (IL-1β, IL-6, IL-12, IL-23, TNF-α, etc.), neutrophil chemokines (CXCL1, CXCL2, CXCL6, CXCL8, etc.), lymphokines (CCL20, CCL3, etc.) and costimulatory molecules, act on dendritic cells, neutrophils and T cells, induce T cell proliferation, neutrophil and Th17 cell recruitment and activation, as well as further secretion of a large number of cytokines, and form a continuously activated inflammatory loop in psoriatic lesions (16). In imiquimod (IMQ)-induced psoriasis mouse models, psoriasis-like pathological changes in systemic as well as KCs-specific IL36R −/− mice were significantly improved compared with wild-type mice, and mRNA levels of cytokines such as IL-17A, IL-17C, IL-22, and IL-23 in lesional skin lesions were also significantly reduced (17).

### 3.2 Epigenetic regulation

Epigenetics refers to stable, heritable changes in gene expression that do not affect DNA sequence changes. Epigenetic mechanisms associated with psoriasis involve DNA methylation, histone modifications, and non-coding RNAs (ncRNAs).

#### 3.2.1 DNA methylation

When DNA methylases (DMNTs) bind a methyl group to the 5′-terminal ends of the cytosine loop of genomic CpG dinucleotides, this is called DNA methylation. Currently, methylation analysis, including genome-wide levels and specific gene loci, has been carried out on the skin of psoriasis patients (18). It was found that genome-wide methylation levels were significantly higher in psoriasis patients’ lesions compared to healthy controls. These levels were also positively related to psoriatic lesion area and severity index (PASI) scores (19). Another genome-wide DNA methylation study of psoriasis patients’ sore skin found 1,108 differentially methylated loci. Twelve of these were linked to genes that help the epidermis differentiate. Treating the skin with TNF-α for one month returned the methylation levels to a normal state (18). Several studies have also shown that psoriasis patients’ skin lesions have differentially methylated gene loci. These gene loci mostly deal with cell proliferation, apoptosis, the cell cycle, and immune system regulation (20). Some genes that were hypermethylated were PDCD5, TIMP2, SELENBP1, CARD14, KAZN, ECE1, MAN1C1, DLGAP4, and SFRP4. Genes that were hypomethylated were S100A9, PTPN22, CYP2S1, EIF2C2, SHP-1, and HLA-DRB1. The levels of methylation were linked to gene expression in a bad way (21). There was a positive correlation between PASI scores and hypermethylation levels of HLA-C, as well as hypomethylation levels of p16 (20). Identification of differentially methylated genes associated with characteristic histopathological changes in psoriasis (e.g., Munro’s microabscesses, epidermal extension, etc.) suggests that DNA methylation may mediate pathological changes in psoriasis (19).

Immune cells were also subjected to DNA methylation analysis. The levels of methylation across the genome in PBMCs from psoriasis patients were much higher than those in healthy controls. The study also found that DNMT1 was highly methylated in PBMCs from psoriasis patients, while MBD2 and MeCP2 were lowly methylated (19). Two research teams reported elevated and decreased genome-wide methylation levels in CD4+ T cells from psoriasis patients, respectively (22, 23). Also, the methylation level of FOXP3 in Tregs cells from people with psoriasis was much higher than that in healthy controls. This meant that FOXP3 expression was lower and there were fewer Tregs cells, which helped psoriasis get worse (24).

#### 3.2.2 Histone acetylation

Lysine acetylation at the amino terminus of histones changes quickly. These changes can cancel out the positive charge of histones, make chromatin structure less rigid, and start transcription all around. Histone H4 acetylation levels were lower in PBMCs from people with psoriasis compared to healthy controls, and acetylation levels were linked to lower PASI scores (18, 23). These results suggest that histone acetylation mechanisms may play a role in psoriasis. Histone acetyltransferases (HATs) and histone deacetylases (HDACs) can catalyze the acetylation and deacetylation of histones, making them potential targets for drug therapy. There was a big rise in the expression of HDAC1 in PBMCs and lesions on the skin of people with psoriasis, but a big drop in the expression of class III HDAC SIRT1 (25). By increasing the expression of FOXP3 and stopping Treg cells from changing into harmful Th17 cells, blocking HDAC can control how Treg cells work (26, 27). In KCs, activated SIRT1 reduces inflammation and speeds up cell death. In a mouse model of psoriasis due to IMQ, resveratrol, a SIRT1 agonist, lessens the severity of lesions and histopathological damage. Phase II clinical trials of the selective SIRT1 agonist SRT2104 have shown its good efficacy and safety in psoriasis (28, 29).

#### 3.2.3 ncRNAs

MicroRNAs (miRNAs) are small single-stranded RNAs that are not protein-coding. More than 250 miRNAs have been found to be abnormally expressed in psoriasis (30–32), and they play an important role in the development of psoriasis by regulating target genes involved in signaling pathways related to KCs proliferation and differentiation, cytokine and chemokine production, and immune inflammatory responses (Table 1). In addition, miRNAs (especially circulating miRNA levels) can also be used as biomarkers for psoriasis diagnosis, monitoring treatment response, and evaluating prognosis (65). Several experiments with cells or animals have shown that controlling certain miRNAs (for example, stopping miR-21, miR-31, or miR-210 and increasing miR-125b, etc.) works well. This suggests that targeting miRNAs could be a new way to treat psoriasis, but more in-depth studies are still needed (66–69).

**Table 1:** Nature products for the treatment of psoriasis.

S. N.	Phytochemicals	Cell/animal model	Method for detecting	Outcomes offered by natural products	References
1	Celastrol	HaCaT and primary human	MTT assay	Increased apoptosis via Bcl-2 attenuation and Bax	(33)
2	Dehydrocostuslactone and costunolide	Primary human Keratinocytes 3D	Annexin-V staining	Inhibited proliferation and inflammation-related genes	(34)
3	Thymol	ovalbumin (Ova)-immunized mice	flow cytometry	The levels of INF γ and IL-17 in CD4 + Tbet + cells were decreased, and the levels of IL-17 in CD4 + Tbet + and CD4 + RORγ T + cells were decreased	(35)
4	Abietic acid	BALB/c mice (Male) BALB/c mice (Male)	flow cytometry flow cytometry	Th17 cell differentiation was inhibited, and TNF-α, IL-17A, TGF-1β, IL-23 levels were down-regulated The proportion of Th17 cells was significantly decreased, and that of Treg cells (CD4+/CD25+/Foxp3+) was significantly increased	(36, 37)
5	Betulinic acid	BALB/c and C57BL/6 mice	Western blot	Inhibited NF-κB signal in skin injury and decreased il-17A production	(38)
6	Paeoniflorin	BALB/c and C57BL/6 mice HaCaT and BALB/c Mice; Psoriatic patients		Th17 cytokine expression was inhibited by affecting STAT3 phosphorylation and RORγ T expression TNF-α-induced chemokine production and leukocyte migration was inhibited by NF-κB and ERK pathways	(39, 40)
7	Resveratrol	Primary culture of human keratinocytes Primary culture of human keratinocytes	MTT assay and hemocytometer cell counting 7-aminoactinomycin D assay	Dose-dependent antiproliferative activity Reduced aquaporin 3 expression	(41)
8	Curcumin	TNF-α-activated HaCaT HaCaT TNF-α-activated HaCaT	Annexin-V staining Nuclei staining Cell counting kit-8 and LDH assay	Apoptosis is the main mechanism for antiproliferation of HaCaT Increased apoptosis by the combination with UVA or visible light Increased apoptosis by the combination with red and blue light	(42–44)
9	Rottlerin	Primary culture of human keratinocytes	MTT assay	Increased apoptosis in an autophagy-dependent pathway	(45, 46)
10	Rhodomyrtone	HaCaT/rabbit Human skin Organ culture/ICR mouse	MTT assay Histology	Elevated apoptosis with no skin irritation Reduced epidermal thickness and hyperplasia	(47)
11	Rhododendrin	Normal human keratinocytes/C57BL/6 mouse	MTT assay	Inhibited proliferation and epidermal thickness	(48)
12	Epigallocatechin-3-gallate	Balb/c mouse	Proliferating Cell Nuclear Antigen	Inhibited epidermal thickness and differentiation regulation	(49)
13	Baicalein	HaCaT	Cell counting	proliferation without affecting ROS production	(50)
14	Delphinidin	Reconstructed psoriatic skin equivalent Flaky skin mice (fsn/fsn)	Ki-67 and PCNA	Inhibited proliferation and inflammation Reduced pathological markers of psoriasiform lesions, infiltration of inflammatory cells, and the mRNA and protein expression of inflammatory cytokines	(51, 52)
15	Amentoflavone	HaCaT/Balb/c mouse	Cell counting kit	Inhibited proliferation and epidermal thickness	(53)
16	Kaempferol	Rat		Reduce PIM1-mediated FOXP3 phosphorylation at S422 and increase FOXP3 expression level in Treg cells	(54)
17	Luteolin	HaCaT and BALB/c mice (Male)		Inhibited the expression of IFN- γ and reduced the promoting effect of IFN-γ; Inhibit HSP90 expression and exosome	(55)
18	Taxifolin	HaCaT and BALB/C mice		Th17 cell differentiation is regulated by inhibition of Notch1 and JAK2/STAT3 signaling pathways	(56)
19	Genistein	Psoriasis-model in mice		causes a reduction of cytokine expressions such as IL-1β, IL-6, TNF-α, CCL2, IL-17, and IL-23	(57)
20	Naringin	Clinical trials on 20 patients		inhibitory effect on TNF-α and IL-6 production	(58)
21	Quercitrin	IMQ-stimulated mice		Reduce levels of TNF-α, IL-6, and IL-17	(59)
22	Amentoflavone	HaCaT		reducing the levels of cyclin D1, IL-17A, and IL-22	(53)
23	Astilbin	BALB/c mice		The differentiation and function of Th17 cells were inhibited by JAK3/STAT3 pathway	(60)
24	Glabridin	IMQ-induced BALB/c mice/HaCaT cells		Inhibited the expression of IL-6, IL-1β, IL-17A, TNF-α, IL-22, and IL-23 and CCL2	(61)
25	Hesperidin	IMQ-induced BALB/c mice/LPS-stimulated HaCaT cells		Modulated the secretion levels of serum leptin, adiponectin, and resistin and inhibited the activation of the IRS-1/erk1/2	(62)
26	Hispidulin	IMQ-induced C57BL/6 J mice/Activated keratinocytes		Alleviated pathologically increased levels of immunoglobulin G2a, myeloperoxidase, and TNF-α, splenic, reduced Th1 and Th17 cell populations, and inhibited gene expression of Th1- and Th17-associated cytokines and chemokines, and phosphorylation of mitogen-activated protein kinases and NF-κB	(63)
27	Berberine	BALB/c mice (Female)		Th17 and Th1 cell differentiation is inhibited by inhibition of JAK2/STAT3 signaling pathway	(64)

Non-protein coding RNAs, or lncRNAs, are a type of RNA that is longer than 200 nucleotides. They change epigenetics, transcription, post-transcription, translation, and post-translation, among other things, to control gene expression at different levels. They do this by interacting with DNA, RNA, or protein through structural interactions and/or complementary base pairing (70). LncRNAs play important regulatory functions in skin physiology, such as KCs differentiation, melanocyte function, hair growth, and wound healing (31). Several studies have found that more than 4,000 lncRNAs are differentially expressed in lesional skin tissues of psoriasis patients by microarray or high-throughput RNA sequencing technology and are associated with KCs differentiation, cell proliferation and apoptosis, and inflammatory immune responses (71–73). Table 1 displays the significant lncRNAs associated with psoriasis. Researchers are still learning more about the relationship between lncRNAs and psoriasis compared to miRNAs. A complete understanding of the functional identification of lncRNAs and their role in the pathogenesis of psoriasis will help with the development of targeted therapeutics.

### 3.3 JAK–STAT pathway

The JAK–STAT pathway contains three main components: the tyrosine kinase-related receptor, JAK kinase, and transcription factor STAT, which can transmit extracellular chemical signals into cells and thus regulate cell growth, differentiation, and response to pathogenic signals. Researchers have identified STAT3, TYK2, IL23A, and IL23R as susceptibility factors for psoriasis (74). After binding to its receptor, IL-23, an important cytokine in psoriasis, promotes the formation of the JAK1/JAK2/TYK2 complex, followed by STAT3/STAT4 phosphorylation and dimerization and translocation into the nucleus, binds to the corresponding target gene promoter, and induces the expression and secretion of cytokines such as IL-17, IL-21, IL-22, and IFN-γ (75). In addition, activated STAT3 promotes the transcription of multiple key genes associated with Th17 cell differentiation, activation, and proliferation. Furthermore, the JAK–STAT pathway also mediates the pathogenic role of other important cytokines in psoriasis. IFN-γ receptor activation induces JAK1/JAK2 activation and dimerization of STAT1, IL-12 receptor activation induces JAK2/TYK2 activation and dimerization of STAT4, thereby promoting Th1 response; IL-4 receptor activation induces JAK1/JAK3 activation and dimerization of STAT6, promoting Th2 response; and IL-6 and IL-21 can activate STAT3 to enhance IL-17 production by Th17 cells and further amplify inflammation (75, 76). Keratinocytes stimulated by IL-22 can activate STAT3 signaling, causing keratinocyte hyperproliferation and inhibiting their differentiation, and show symptoms of acanthosis and skin inflammation in mouse models (77). It has also been found that activation of STAT3 inhibits differentiation and promotes the proliferation and secretion of antimicrobial peptides in KCs, and psoriasis-related symptoms occur in transgenic mice overexpressing STAT3 (78). The expression of JAK1 is positively correlated with the PASI score, and JAK1 and STAT3 tissue expression can be used as markers of psoriasis severity (78, 79). Therefore, the JAK–STAT signaling pathway is one of the important targets for psoriasis drug development. Researchers have tested several JAK inhibitors in plaque psoriasis, demonstrating their good efficacy. Additionally, two phase III randomized controlled clinical trials have shown that treatment with tofacitinib at 5 and 10 mg twice daily significantly reduces PASI 75 by up to 75%, with improvement observed in a dose-dependent manner (80).

### 3.4 Aryl hydrocarbon receptor

AhR is a class of intracellular ligand-activated receptors and transcription factors that are widely expressed in the skin and can sense a variety of endogenous and exogenous ligand molecules and play an important role in maintaining skin homeostasis by regulating environmental toxin metabolism, light-induced response, oxidative stress, KCs differentiation, skin barrier function, pigmentation, and the skin immune network (81). Whether AhR activation produces deleterious or beneficial biological effects depends on the skin state (health or inflammation), and the source or nature of the ligand has been demonstrated (81). In psoriasis patients and mouse psoriasis models, Th17 cells highly express AhR, and IL-22 production by Th17 cells has been found to be dependent on AhR (82, 83). This suggests that AhR is closely associated with cytokine secretion by Th17. Furthermore, researchers have detected overexpression of AhR in lesional KCs from psoriasis patients (84). These lines of evidence indicate that AhR signaling is overactivated in psoriasis. In a mouse model that was given IMQ, however, knocking down AhR causes IL-22, IL-17, and IL-23 gene expression to rise, which makes skin inflammation worse (85). A shot of 6-formylindolo [3,2-B] carbazole (FICZ), which is an AhR receptor agonist, given intraperitoneally helped psoriatic mice feel better (86). AhR can also regulate the expression of multiple terminal differentiation proteins such as filaggrin (FLG) and methyptilin (LOR) in KCs, and the use of endogenous AhR ligand agonists can up-regulate the expression of FLG and LOR, so the activation of AhR contributes to the differentiation and maturation of KCs (82, 87, 88). This suggests that the AhR signaling pathway may play a role as a “double-edged sword” in psoriasis, and in-depth exploration of its role is conducive to the clinical diagnosis and treatment of psoriasis.

### 3.5 Rho-associated kinase

RCK is a serine/threonine kinase that can be activated by Rho GTPase and then phosphorylate its substrates to play a biological role. It is part of the AGC protein kinase family and has two isoforms, ROCK1 and ROCK2. Inhibition of ROCK2 can block the role of IL-23/Th17 in psoriasis. Recent studies have found that ROCK2 plays an important role in regulating autoimmunity and inflammation. In CD4+ T cells from healthy volunteers, antagonizing ROCK2 using inhibitors or siRNA reduced IL-17 and IL-21 secretion by inhibiting transcription factors STAT3, IRF4, and RORγt (89). In a phase II clinical trial of the ROCK2 inhibitor KD025, it lowered the levels of IL-17 and IL-23 in the blood of people with psoriasis. It also decreased the thickness of the skin, the expression of keratin 16, and the number of T cells that came into the lesion site. It also improved PASI scores (90).

### 3.6 Sphingosine-1-phosphate

S1P is a bioactive lipid produced by sphingomyelin metabolism catalyzed by sphingosine kinase (SPHK), which can act as an intracellular second messenger or be secreted into the extracellular space, bind to the S1P receptor (S1PR) on the cell membrane, and regulate biological processes such as cell proliferation, differentiation, apoptosis, angiogenesis, and inflammatory response by activating downstream signaling pathways (77). Psoriasis patients showed increased expression of S1P in their serum, while treatment with biologics did not decrease the circulating level of S1P (91). The role of this signaling pathway in psoriasis requires further investigation. Liao et al. discovered that S1P could cause Th17 cells to differentiate. On the other hand, S1P could stop KC cells from multiplying and cause them to differentiate. It could also stop KC cells from expressing cyclin and increase markers of early and late differentiation in KC cells (92–94). In addition, S1P is also able to transiently increase Ca2+ concentration by activating S1PR3, which is essential for the differentiation of KCs (95). This stops the growth of KCs by keeping the ERK protein active for longer and temporarily turning off the Akt protein (96). Giving S1P directly to the skin of the IMQ mouse model reduced inflammation and excessive skin growth, which suggests that it might help with psoriasis. However, further studies are needed to understand why S1P circulating levels are increased in psoriasis patients and how S1P regulates KCs and immune cells differently (97).

### 3.7 Metabolism abnormal

Abnormal glucose metabolism and excessive proliferation of KCs in psoriasis are like those in tumor cells; glucose uptake is increased, and glycolytic levels are significantly increased, which produce many ATPs and intermediates and promote KCs proliferation. The levels of glucose transporter 1 (GLUT1) and M2 pyruvate kinase (PKM2) are significantly higher in the skin lesions of psoriasis patients and IMQ-induced mouse models. Blocking GLUT1 or PKM2 has been shown to stop the growth of KCs (98). Also, in a model of psoriasis caused by IMQ, using GLUT1 knockout mice or drug inhibitors, along with PKM2 inhibitors or glycolytic inhibitors such as 2-deoxy-D-glucose (2-DG), greatly decreased the development of psoriasis-like skin growths and lesions (99, 100).

New studies have also shown that the metabolism of amino acids is off in people with psoriasis, and in KCs, the psoriasis autoantigens are mostly caused by the metabolism of arginine being off (101). In addition, L-amino acid transporter 1 (LAT1) expression levels were significantly increased in T cells from lesional skin lesions of psoriasis patients and IMQ-induced mouse models, and LAT1 promoted the proliferation of IL-17+ γδ T cells and Th17 cells and the secretion of IL-17 and IL-22 through the PI3K/Akt/mTOR pathway, while the LAT1 inhibitor JPH203 significantly improved IMQ-induced psoriasiform lesions in mice (102). These findings indicate that targeting abnormal metabolism could be a novel therapeutic approach for treating psoriasis.

### 3.8 Circadian rhythm

Circadian rhythm regulates the state and function of the skin. With the disclosure of the mechanism of circadian rhythm regulation, recent studies have reported that the occurrence of psoriasis is associated with circadian rhythm. According to epidemiological studies, psoriasis is more likely to happen when the circadian rhythm is thrown off. This is also true for the release of cytokines like IL-17, IL-6, and IL-1β, which are closely linked to the development of psoriasis (103). In addition, changes in circadian rhythm-regulated gene expression were detected in both the lesional and non-lesional skin of psoriasis patients. The clock genes REV-ERB and ROR are important for their anti-inflammatory and immune-modulatory effects. Synthetic REV-ERB antagonists can stop γε T cells from making IL-17, which makes psoriasis symptoms go away in mouse models (104). Further studies are needed to elucidate the association between circadian rhythm and psoriasis, as it represents a new direction in psoriasis research.

## 4 Targeted therapy of psoriasis with natural products

Currently, there is no definitive treatment for psoriasis; however, specific interventions have shown encouraging results (105–107). People with moderate-to-severe psoriasis who were treated with monoclonal antibodies like infliximab, ixekizumab, risankizumab, bimekizumab, guselkumab, secukinumab, and brodalumab had great results (108). However, prolonged use of these medications is frequently associated with the development of various adverse effects (108, 109). Consequently, natural products and traditional and alternative remedies are gaining increasing scientific attention. Bioactive compounds can be found in large amounts in natural products. In fact, many studies have shown that some natural extracts have anti-inflammatory, antiproliferative, and antioxidant properties (110, 111). These pieces of evidence illustrate their potential utility in the treatment of various diseases, including psoriasis, owing to their safety profile and the likelihood of improved patient compliance (112–114). In the following part, we summarize the natural products for the treatment of psoriasis.

### 4.1 Terpenoids

Terpenoids, which are also called isoprenoids, are made up of compounds and their derivatives that come from metronomic acid and have the isoprene unit as their main building block. This unit has five carbon atoms. Examples of oxygenated derivatives include esters, alcohols, aldehydes, ketones, and carboxylic acids. Terpenoids are abundant in the natural world and serve as the primary constituents of specific plant-based fragrances, resins, and pigments. Rose oil, eucalyptus oil, and turpentine are among the included substances. Furthermore, terpenoids also include certain animal hormones and vitamins (115).

#### 4.1.1 Celastrol

Celastrol, a chemical compound, is obtained from the root bark of Tripterygium wilfordii Hook F, a botanical species recognized for its various biological activities in traditional Chinese medicine. Celastrol is a triterpene isolated from *Celastrus orbiculatus*. In Chinese medicine, the extract of *C. orbiculatus* has been utilized for centuries to treat rheumatoid arthritis, cutaneous diseases, and bacterial infections. Zhou et al. evaluated the antiproliferative effect of celastrol on HaCaT cells and primary human keratinocytes (116). The growth of HaCaT and primary keratinocytes was inhibited by celastrol in a concentration-dependent manner, as evidenced by its IC50 values of 1.1 and 2.9 μM, respectively. The IC50 for fibroblasts was 6.8 μM. Celastrol induced a notable decrease in the expression of pro-apoptotic Bcl-2 (Bax) and an increase in the expression of anti-apoptotic Bcl-2, indicating that both death-receptor and mitochondrial pathways are implicated in the apoptotic mechanism (117). The findings from this experiment indicate that celastrol, a naturally occurring compound, possesses potential and should be further explored for its potential as a pharmaceutical treatment for psoriasis.

#### 4.1.2 Dehydrocostuslactone and costunolide

Dehydrocostuslactone and costunolide are sesquiterpene lactones that were isolated from certain plants, such as Magnolia sieboldii. Both compounds are effective in mitigating inflammation and exerting pro-apoptotic activity (33, 118). Scarponi et al. investigated the impact of both naturally occurring lactones on cytokine-induced inflammatory regulation and keratinocyte proliferation (34). While the chemicals increased cell cycle arrest and death, the lactones inhibited proliferation and gene expression associated with cell cycle progression (34). Dehydrocostuslactone and costunolide decreased the inflammatory and regulatory genes in IL-22-activated keratinocytes, including C-C motif chemokine Ligand 2 (CCL2), C-X-C motif chemokine 10 (CXCL10), and intercellular adhesion molecule 1 (ICAM-1). These findings encourage the further application of psoriasis therapy.

#### 4.1.3 Thymol

Thymol is the main monoterpene phenol present in essential oils, which are isolated from plants belonging to the Labiatae family as well as from other plants such as Verbenaceae, Scrophularia, and Ranunculaceae (119). Thymol has antioxidant, anti-inflammatory, antibacterial, and antifungal effects (119). In *in vitro* and *in vivo* experiments, Namdari et al. demonstrated that thymol promoted the differentiation of naïve CD4 + CD25- T cells into CD4 + CD25 + Foxp3 + Tregs [66.9–71.8% vs. control (47%)] and increased the intensity of Foxp3 expression on Treg cells (35). Furthermore, flow cytometry assessment showed that CD4 + Foxp3 + Treg increased in the spleen of thymol-treated Ovaimmunized mice, and CD4 + RORγt+Th17 cell levels decreased, resulting in significant decreased Th1/Treg and Th17/Treg ratios.

#### 4.1.4 Abietic acid

Abietic acid is a diterpenoid compound that is taken from Pimenta racemosavar. Grissea. It stops cell growth, kills bacteria, and fights obesity. According to Li et al., oral administration of abietic acid reduces PASI scores, modifies the balance of Th17/Treg cells in the mouse spleen, and downregulates serum cytokines like TNF-α, IL-17A, TGF-1β, and IL-23 to alleviate IMQ-induced psoriasis-like skin inflammation (36, 37). In addition, 16S rRNA gene sequencing showed that the Abietic acid group mice had higher genus levels of Kurthia, Citrobacter, and Klebsiella but lower genus levels of gut bacteria linked to inflammation, such as Anaerotruncus and Christensenella. Furthermore, the correlation analysis demonstrated a strong association between the key microbiota and the inflammatory indexes related to psoriasis-like conditions (36, 37).

#### 4.1.5 Betulinic acid

Betulinic acid, also known as 3-hydroxy-lup-20(29)-en-28-oic acid, is a naturally occurring pentacyclic triterpenoid derived from a variety of plants, including the birch tree, birch bark oil (betulae pil), and paeoniaceae. Betulinic acid has been documented to exhibit a multitude of biological properties, such as antioxidant, anti-inflammatory, anti-cancer, anti-fibrotic, and anti-angiogenic effects. Recent studies have documented that betulinic acid inhibits the synthesis of several pro-inflammatory mediators and cytokines. Betulinic acid ameliorated an IMQ-induced psoriasis-like skin lesion in rodents by inhibiting the development of Th17 and IL-17+ γδ T cells, according to Liu’s research (38). In addition, betulinic acid inhibited the gene expression of pro-inflammatory mediators, including RORγt, IL-17A, IL-6, and TNFα, in the cutaneous lesion. Significantly, it additionally inhibited NFκB signaling in the dermal tissue of the psoriatic animals. Betulinic acid ultimately inhibited the proliferation of T cells and the production of IL-17A by CD4+ T cells *in vitro*. Therefore, betulinic acid has the potential to serve as a natural anti-psoriatic medication for the treatment of psoriasis in humans.

#### 4.1.6 Paeoniflorin

Paeoniflorin constitutes a significant proportion (over 90%) of the bioactive constituents found in the total glucosides of paeony. It finds extensive application in China as a therapeutic agent for inflammatory and autoimmune disorders, including rheumatoid arthritis, psoriasis, Sjogren’s syndrome, and systemic lupus erythematosus (120). One study demonstrated that paeoniflorin could mitigate the skin injury caused by IMQ-induced inflammation in mice with psoriatic model disease (39). Following further examination, it was determined that paeoniflorin reduced the quantity of F4/80 + CD68+ macrophages and CD11b + Gr-1+ neutrophils in the skin of mice challenged with IMQ, as well as the cytokine production associated with these cells (TNF-α, IL-1β, IL-6, IL-12, IL-23), inducible nitric oxide synthase (iNOS), and macrophage inflammatory protein-2 (MIP-2) (39).Furthermore, paeoniflorin also down-regulated Th1/Th17-associated cytokine production (39). In mice with psoriasis-like skin lesions induced by IMQ, paeoniflorin can substantially ameliorate the condition by inhibiting epidermal cell proliferation, infiltration of inflammatory cells, and abnormal differentiation. The expression of Th17 cytokines can be inhibited *in vitro* and in mice by paeoniflorin. By modulating Stat3 phosphorylation and RORγt expression, PF might exert its effect (40).

### 4.2 Polyphenols and phenolic compounds

A class of chemical elements known as polyphenols is classified as such because they contain multiple phenolic groups. The primary sources of natural polyphenols are alcohol, fruits and vegetables, nuts, soybeans, tea, and cacao. “The seventh category of nutrients” are polyphenols, which possess antioxidant properties (121).

#### 4.2.1 Resveratrol

Resveratrol is a non-flavonoid polyphenol organic compound. Resveratrol has been demonstrated to possess antioxidant, anti-inflammatory, anticancer, and cardiovascular protective properties in both *in vitro* and animal studies (41). The antiproliferative activity of resveratrol against primary keratinocytes was investigated by Holian and Walter (41). Resveratrol inhibited proliferation in a concentration- and time-dependent manner. By counting the cells of the hemocytomer, the IC50 was determined to be 0.5 M. The inhibitory impact on cellular proliferation was a result of resveratrol’s modulation of the cellular redox state (41). The role of aquaporin 3 in the antiproliferative mechanism of resveratrol was further elucidated by Wu et al. (41). Aquaporin 3 is a water-transporting protein that is expressed in epidermal keratinocytes. The overexpression of aquaporin 3 leads to keratinocyte proliferation and epidermal thickening (122). The nontoxic concentrations (<40 μM) of resveratrol restrained the proliferation of the primary culture of neonatal human keratinocytes. Resveratrol at 40 μM significantly decreased the aquaporin 3 mRNA level by >5-fold. This compound also inhibited the phosphorylation of extracellular signal-regulated kinase (ERK).

#### 4.2.2 Curcumin

Curcumin, which is a diketone and a natural phenolic antioxidant, is extracted from the rhizomes of *Curcuma longa*, Curcuma zingiberensis, mustard, curry, and *Curcuma longa*. It possesses aromatic and unsaturated aliphatic groups. Curcumin has demonstrated therapeutic properties, and humans can safely consume up to 8 g of it daily without experiencing any adverse effects (123). Some evidence suggests that curcumin may have the ability to alleviate psoriasis (124). Sun et al. investigated the impact of curcumin on the apoptosis of TNF-α-activated HaCaT cells (42). The upregulation of anti-apoptotic proteins (IAP), IAP2, and B-cell lymphoma-extra-large (Bcl-xL) was observed in response to TNF-α, whereas the inhibition of these proteins was induced by 7.37 μg/mL curcumin. Curcumin also inhibited TNF-α-activated NF-κB, IL-6, and IL-8. The antiproliferative ability of resveratrol can be enhanced in combination with light irradiation. The HaCaT cells were pretreated with curcumin at 0.1 ~ 1 μg/mL for 1 h and then irradiated with UVA or visible light (43). The result revealed that the combination of curcumin (1 μg/mL) and UVA (1 J/cm2) induced 40% of the cells to have apoptotic nuclei. Niu et al. investigated the combination of curcumin, red light (630 nm), and blue light (405 nm) for attenuating the proliferation of TNF-α-activated HaCaT, a simulation of psoriasis lesions (44). The curcumin concentration at 0.16 ~ 2.5 μM showed no proliferation inhibition on keratinocytes (44). A significant inhibition was observed in the presence of 0.16 and 0.62 μM curcumin when it was combined with red light and blue light, respectively (44). Light in isolation had no discernible impact on proliferation. This finding suggested that the apoptosis of keratinocytes treated with curcumin was enhanced upon exposure to light. The combined treatment inhibited NF-κB activity and stimulated caspases 8 and 9 with the preservation of cell-membrane integrity (44).

#### 4.2.3 Rottlerin

Rottlerin is a polyphenol that is purified from Mallotus phillippinensis. It has been reported that this compound possesses antihypertensive, antifertility, and antiallergic properties (125). Rottlerin exhibits significant inhibitory effects on keratinocyte proliferation by inhibiting the upregulation of NF-κB induced by both basal and hydrogen peroxide (45). The inhibitory effect of rottlerin on primary keratinocyte proliferation was investigated by Min et al. (46). After exposure to rottlerin at concentrations of 5 and 10 μM, the apoptotic percentage of keratinocytes was observed to be 27 and 56%, respectively. Significant reductions in the levels of TNF-α, IL-6, and IL-23 were observed in keratinocytes activated by TPA after treatment with rottlerin. In addition to alleviating the IMQ-stimulated psoriasiform lesion, oral rottlerin inhibited the proliferation of keratinocytes, the infiltration of immune cells, and the proliferation of blood vessels. In addition to inhibiting the proliferation of keratinocytes (HaCaT and NHEKs cells) and inducing their apoptosis, Min et al. demonstrated that rottlerin significantly obstructs the secretion of psoriasis-promoting cytokines (TNF-, IL-6, IL-17, IL-22, and IL-23) at concentrations of 0 μM, 1 μM, 5 μM, and 10 μM (45).

#### 4.2.4 Rhodomyrtone

Rhodomyrtone is the main active ingredient in *R. tomentosa* extract. It has antibacterial, anti-inflammatory, and immunomodulatory properties (126). The potential impact of rhodomyrtone on the proliferation, growth arrest, and apoptosis of HaCaT was examined by Chorachoo et al. (47). After being incubated with rhodomyrtone at 2–32 μg/mL for 24 h, 13.6 to 61.6% of HaCaT showed antiproliferative activity. In the scratching assay, the application of rhodomyrtone at a concentration of 2 μg/mL caused a 61.8% delay in the closure of the lesion. Chromatin condensation and fragmentation of nuclei were observed after treatment with rhodomyrtone. The flow cytometry analysis revealed that the proportion of azotosomes was greater in the experimental group that underwent apoptosis than the control group. An *in vivo* skin irritation test conducted on a rabbit following rhodomyrtone administration revealed the absence of erythema and edema. The inhibitory effect of rhodomyrtone on TNF-α/IL-17A-driven inflammation was further rated. Rhodomyrtone decreased the inflammatory gene expression in the human skin organ culture. This molecule also inhibited TNF-α-activated ERK, JNK, and p38 phosphorylation. Putting rhodomyrtone (0.18 and 0.64 mg/cm2) on a mouse’s psoriasiform lesion caused by IMQ reduced the thickness and hyperplasia of the epidermis. Taken together, both antiproliferation and anti-inflammation are the therapeutic mechanisms for psoriasis inhibition by rhodomyrtone.

#### 4.2.5 Rhododendrin

Rhododendrin is an arylbutanoid glycoside that was isolated from *Rhododendron brachycarpum*. It is an inhibitor of inflammation for use in treating inflammatory skin diseases (48). Jeon et al. defined in detail the mechanism of action of this compound and its potential as a psoriasis treatment (127). The study conducted on keratinocytes *in vitro* revealed that rhododendrin inhibited the toll-like receptor (TLR)-7, NF-κB, and MAPK signaling pathways. Caveolin-1 loss in keratinocytes contributes to psoriasis development. The preservation of caveolin-1 expression with TLR-7 following rhododendrin treatment suggested that this compound plays a crucial role in the maintenance of skin homeostasis. Rhododendrin applied topically reduced IMQ-induced immune-cell infiltration, hyperplasia, cytokines (TNF-, IL-1, IL-6, IL-8, IL-17, and IL-23), and immune-cell infiltration by a significant margin.

#### 4.2.6 Epigallocatechin-3-gallate

A tea polyphenol called epigallocatechin-3-gallate (EGCG) may help keratinocytes differentiate normally and lower the risk of cancer linked to PUVA (49). Zhang et al. looked at how EGCG might stop psoriasiform lesions from getting worse when IMQ is added (49). Six days of topical EGCG administration resulted in a reduction in epidermal thickness from 70–150 to 30–80 μm. This phenomenon may be attributed to EGCG’s capacity to decrease PCNA expression while increasing caspase 14 levels. Caspase 14 primarily regulates the formation of normal barriers and terminal differentiation in keratinocytes.

### 4.3 Flavonoids

Flavonoid proto-compounds refer to a general term for a class of compounds derived with 2-phenylchromogen ketone as the backbone. It now refers to the general term for a series of compounds formed by the interconnection of two benzene rings through three carbon atoms, that is, a general term for a class of compounds with a C6-C3-C6 structure. Flavonoids are widely present in plants in nature and belong to plant secondary metabolites.

#### 4.3.1 Baicalein

Baicalein is a natural yellow pigment that is a diterpenoid compound with a variety of biological activities. Baicalein has a structural formula of C15H10O2, and its molecular weight is 222.24 g/mol. Because baicalein is mainly found in Scutellaria baicalensis, it is named. Baicalein, an additional flavonoid, controls the proliferation and differentiation of keratinocytes (50). Baicalein (10 μM) exhibited a marginal inhibitory effect on HaCaT proliferation while leaving ROS production, cytochrome c, and apoptosis unaffected. Treatment with baicalein increased the proportion of cells in the G0/G1 phase from 60 to 70%.

#### 4.3.2 Delphinidin

Delphinidin is an anthocyanidin that functions as an antioxidant and provides the primary plant pigment. As it regulates keratinocyte differentiation and inhibits inflammation, delphinidin has been identified as a potential psoriasis treatment (128). In their study, Hasan Mukhtar et al. investigated the effect of delphinidin on a full-thickness 3D reconstituted human skin model of psoriasis [psoriatic skin equivalent] as opposed to a normal 3D human skin equivalent to determine whether it could induce differentiation and inhibit the expression of proliferation and inflammation markers (51). The research demonstrated that treatment with delphinidin in PSE promotes differentiation while inhibiting proliferation and inflammation, thereby enabling total skin regeneration. In addition, their findings indicate that delphinidin is superior to all-trans retinoic acid and at least as effective as Vit-D3 when compared to its effect in comparison to all-trans retinoic acid (51). In their investigation, F. Afaq et al. assess whether the topical administration of delphinidin can influence pathological indicators of psoriasiform lesions in rodents with flaky skin, as well as whether this modulation is accompanied by enhanced epidermal differentiation, decreased proliferation, and inflammation (52). Five times per week, for a maximum of fourteen weeks, delphinidin (0.5 mg per cm2 and 1 mg per cm2 skin areas, respectively) was applied topically to fsn/fsn female homozygous flaky skin mice (five weeks old) (52). A decrease in the development of psoriasiform lesions was observed in rodents with flaky skin following the topical administration of delphinidin (52). In addition to impeding keratinocyte proliferation and inducing keratinocyte differentiation, delphinidin treatment modulated the expression of tight junction proteins (52). In mice with flaky skin, delphinidin treatment additionally increased the expression of AP-1 proteins and inhibited the infiltration of macrophages and neutrophils, which are proinflammatory cytokines (52).

#### 4.3.3 Amentoflavone

Amentoflavone is a natural bioflavonoid with many biological activities, including anti-inflammatory, antioxidant, and neuroprotective effects. By lowering the levels of cyclin D1, IL-17A, and IL-22 in HaCaT cells that had been treated with cytokines, this bioflavonoid compound stopped cell growth and caused programmed cell death (apoptosis). The oral amentoflavone dosage of 50 mg/kg reduced the epidermal thickness of the IMQ-induced psoriasis *in vivo* model from about 300 to 180 m. The chemical exhibited a substantial decrease in the expression of several cytokines (IL-17A, IL-22, and IL-23) in the lesion (53).

#### 4.3.4 Kaempferol

Kaempferol is a natural flavonoid dietary substance and polyphenolic antioxidant present in a variety of fruits and vegetables. A study showed that the administration of kaempferol effectively shielded mice from developing psoriasis-like skin lesions caused by the topical application of IMQ (54). Kaempferol decreased the infiltration of CD3+ T cells and suppressed the expression of key proinflammatory cytokines, such as interleukin (IL)-6, IL-17A, and tumor necrosis factor (TNF)-α, in the psoriatic skin lesion (54). Additionally, it suppressed the activity of proinflammatory nuclear factor kappa B (NF-κB) signaling in the skin (54). The therapeutic results were linked to a notable rise in the frequency of CD4+ forkhead box protein 3 (FoxP3) + regulatory T cells (Tregs) in the spleen and lymph nodes, as well as the presence of FoxP3-positive staining in the skin lesion (54). In contrast, the reduction of CD4 + CD25+ Tregs resulted in the reversal of the therapeutic benefits of kaempferol on the skin lesion (54). Kaempferol also reduced the proportion of IL-17A + CD4+ T cells in the spleen and lymph nodes of mice with psoriasis caused by IMQ (54).

#### 4.3.5 Luteolin

Luteolin, mostly in the form of glycosides, is present in a variety of plants, which are highly abundant in green orchid, pepper, wild chrysanthemum, honeysuckle, and perilla. It has antitussive and expectorant effects. According to the latest study, respiratory symptoms such as cough, sputum, and wheezing are associated with chronic airway inflammation. Ping Li et al. demonstrated that luteolin can relieve the lesions and symptoms of psoriasis by reversing the effect of IFN-γ, inhibiting the expression of HSP90 and exosome secretion, and regulating the proportion of immune cells through network pharmacology combined with *in vitro* and *in vivo* experimental validation (55). The precise outcomes are as follows: Based on a pharmacology network analysis, luteolin is anticipated to have a molecular-target correlation with HSP90, making it a potential chemical of interest (55). Cell tests demonstrated a strong correlation between the development of psoriasis and elevated levels of IFN-γ (55). This rise in IFN-γ stimulated the transcriptional expression and exosome secretion of HSP90 in HaCaT cells (55). Conversely, luteolin suppressed these effects and mitigated the promotion of IFN-γ (55). Luteolin had a slightly lesser impact on HSP90 compared to INF-γ (55). Animal investigations have shown that luteolin and 17-AAG have comparable effectiveness in reducing skin tissue lesions and symptoms (55). Additionally, they enhanced the expression of Hsp90 mRNA and protein in skin tissue and stimulated the release of Hsp90 exosomes in plasma (55). Luteolin decreased the ratio of Th1/Th2 and Th17/Treg in the immune cells of mice with psoriasis and suppressed the elevation of Th1 and Th17 in the peripheral blood (55). In another study, Zuyi Weng examined the effects of luteolin on the activation of human cultured keratinocytes (129). Researchers have found that luteolin effectively stops the production of IL-6, IL-8, and VEGF by TNF. It also stops the growth of human keratinocytes (129). Luteolin also lowers the activation of the transcription factor NF-kB by TNF at both the gene and protein levels. Elevated mRNA expression of RELA, the gene encoding the NF-kB p65 subunit, is observed in human skin (129). Their results suggest that luteolin is a promising candidate for development into an effective treatment for inflammatory skin conditions, including Ps, which is characterized by keratinocyte hyperproliferation and chronic inflammation (129).

#### 4.3.6 Taxifolin

Taxifolin, usually referred to as dihydroquercetin, is a prevalent flavonoid that is frequently present in Pinaceae plants, including *Pseudotsuga taxifolia*, Taxus chinensis, Pinus roxburghii, and *Cedrus deodara* (130). It can also be extracted from other plants such as rhododendrons, *smilax China* l, and milk thistle. Taxifolin exhibits a diverse range of pharmacological properties, including anti-cancer, antioxidant, anti-inflammatory, anti-proliferative, and antibacterial effects (130). Furthermore, taxifolin has been employed in clinical settings for the management of cardiovascular and cerebrovascular disorders (130). In their study, Yuan et al. discovered that taxifolin effectively suppressed the excessive growth caused by lipopolysaccharide (LPS) in Hacat cell lines (130). Additionally, taxifolin has shown a substantial improvement in alleviating psoriasis produced by imiquimod (IMQ) in BALB/c mice as compared to the control group (56). TXL does not have a notable impact on the overall proportion of T cells in the lymph nodes that drain the skin (SDLN). However, it does decrease the number of pro-inflammatory Th1 and Th17 cells in both skin lesions and SDLN (56). Taxifolin can control the development of T-helper cells by blocking the Notch1 and JAK2/STAT3 pathways (56). This suggests that TXL could be used as a treatment for psoriasis.

#### 4.3.7 Genistein

Genistein is a flavonoid often present in several crops, including soybeans and fava beans. The concentration of it in food ranges from 1 to 2 mg/g (131). Multiple studies have revealed a wide range of biological effects, including antioxidant, antiangiogenic, and anticancer activities. Wang et al. found that giving genistein to a mouse model of psoriasis at doses of 50 and 100 μM for two hours lowers the production of cytokines like IL-1β, IL-6, TNF-α, CCL2, IL-17, and IL-23 (57). Genistein also lowered the phosphorylation of STAT3 and stopped the phosphorylation of Iβ. It also stopped the translocation of nuclear NF-alpha in both IMQ-treated mouse skin and HaCaT cells that were stimulated with TNF-alpha (57). Smoli’nska et al. found through laboratory experiments that genistein, at a concentration of 1 μg/mL, suppressed the production of reactive oxygen species (ROS) in HaCaT cells. TNF- or LPS activated these cells, and they underwent a 24-h incubation period. Research shows that genistein can lower the production of inflammatory cytokines that depend on NF-κB in keratinocytes that are activated by TNF or LPS in a model of psoriasis. NF-κB is activated by reactive oxygen species (ROS).

#### 4.3.8 Naringin

Naringin is most found in citrus plants like grapefruit, orange, or cooked tomato paste, cherries, beans, and oregano (132). This flavonoid offers several therapeutic benefits linked with psoriasis etiology, including anti-inflammatory action and chemokine production inhibition. In clinical studies with 20 patients, Deenonpoe et al. found that naringin stopped the production of TNF- and IL-6. High levels of these cytokines have immune system effects in psoriasis (58).

#### 4.3.9 Quercetin

Quercitrin is a flavonoid monomer compound widely present in plants and has various pharmacological effects such as anti-oxidation, anti-tumor, hypoglycemic, and hypolipidemic effects (133). Quercitrin has some anti-inflammatory activity, mainly manifested as the inhibition of proinflammatory factors. In various cell cultures, including MOLT-4 cells, leukemia cells, and MCF-7 cells, quercetin triggered apoptosis via ROS production. ROS-related p53 protein ubiquitination caused keratinocyte death when arsenic trioxide and quercetin were combined. Apoptotic indicators such as caspase-3, DNA ladders, and poly (ADP-ribose) polymerase (PARP) were enhanced when mitochondrial membrane potential was decreased. However, the use of arsenic for psoriasis treatment may be hampered by the risk of arsenic poisoning leading to skin cancer. Chen et al. investigated the effect of quercetin on cytokine levels in dosages of 30, 60, and 120 mg/kg for 7 days. They found that after administering different doses of quercetin to psoriatic models and IMQ-stimulated mice, the levels of TNF, IL-6, and IL-17 were dramatically reduced (134). To enhance the stability and solubility of quercetin, Paleco et al. synthesized lipid microparticles and mixed them with microneedles to create a more permeable compound. Using microneedles to apply free quercetin to the skin did not increase its permeability. Although microneedles can effectively penetrate the skin and access deeper layers, quercetin’s weak solubility limits its permeability (59). The combination of microneedles with lipid microparticles, on the other hand, significantly improves permeability. After microneedle therapy, lipid microparticles localised in the SC and viable epidermis rose by 2 and 5 times, respectively, compared to untreated skin. This is explained by the fact that the microparticles can enter the epidermis via microconductors created by the microneedles but do not disseminate to the deeper layers of skin. These findings suggest that the combination of quercetin lipid particles with microneedles has a specific epidermal targeting effect. Therefore, quercetin mixed with microneedles can be used to treat psoriasis by reducing the expression of IL-17 and TNF-β, thereby reducing inflammatory cell infiltration in the epidermis (59).

#### 4.3.10 Amentoflavone

Amentoflavone is a biflavonoid with anti-inflammatory and antioxidant properties (135). The prospect of using amentoflavone to treat psoriasis was investigated. By lowering the levels of cyclin D1, IL-17A, and IL-22, this biflavonoid stopped cytokine-stimulated HaCaT cells from multiplying and caused them to die. Oral amentoflavone (50 mg/kg) lowered the thickness of the epidermis from about 300 m to 180 m in a mouse model of psoriasis caused by IMQ (53). The substance greatly reduced the levels of several cytokines (IL-17A, IL-22, and IL-23) in the lesion (53).

#### 4.3.11 Astilbin

Astilbin, a bioactive compound found in large amounts in the rhizome of Smilax glabra and other plants, has been used to treat psoriasis in the past (136). Multiple bioactivities of clinical significance have been attributed to aspirin, including antioxidant and anti-inflammatory properties. According to what Di et al. found, asstilbin stopped inflammation and keratinocyte overproliferation in a mouse model of imiquimod-induced psoriasis. *In vitro*, aflatilbin inhibited IL-17 secretion and decreased Th17 cell differentiation in a dose-dependent fashion (60).

#### 4.3.12 Glabridin

Glabridin is a flavonoid extracted from a precious plant called *Glycyrrhiza glabra*, which is known as ‘whitening gold’ because of its strong whitening effect, can eliminate free radicals and melanin at the muscle base, and is a whitening and anti-aging holy object of the skin. It is also used as a natural flavoring, food additive, and beer lavor. Studies have shown that glabridin can reduce inflammation by lowering the levels of pro-inflammatory cytokines, nitric oxide (NO), and reactive oxygen species (ROS) (61). Penghui Li did research to look into the possible positive effects and possible underlying mechanisms of action of glabridin on HaCaT cells and a mouse model that was given psoriasis-like symptoms using IMQ (61). When glabridin was added to HaCaT cells that had been stimulated with LPS, the levels of iNOS, p65, IL-6, and IL-1β were found to go down. IL17A, IL-22, and IL-23 levels induced by TNF-α also decreased in a dose-dependent fashion when glabridin was administered. According to these findings (61), glabridin presumably inhibited pro-inflammatory cytokines and mediators linked to psoriasis. Histological analysis also showed that Glab treatment improved psoriasis-like symptoms by making the epidermis smoother, lowering the number of inflammatory cells that entered, and lowering the thickness of the epidermis (61). It was reported that pro-inflammatory cytokines and mediators such as IL-17A, IL-22, IL-23, IL-6, IL-1β, and p65 play an important role in the development and maintenance of psoriasis (137–140). The findings revealed that p65, IL-17A, IL-22, IL-23, IL-6, IL-1β, and IL-17A were all decreased in glabridin-pretreated animals. This suggested that the topical administration of glabridin could potentially ameliorate the inflammatory condition of the animals.

#### 4.3.13 Hesperidin

Hesperidin, which is present in the peels of tangerines, is utilized as a component in Chinese herbal remedies. Hesperidin, a flavone, is chemically linked to the disaccharide retinues, forming a flavanone glycoside. The diverse physiological qualities of hesperidin encompass anti-inflammatory, anticancer, and the reduction of obesity, hyperglycemia, and hyperlipidemia (62). According to Y. Wang’s research, hesperidin has been shown to effectively reduce symptoms of psoriasis-like thickness, skin scaling, and erythema caused by IMQ. The therapeutic efficacy of hesperidin for mice was comparable to that of MTX. Hesperidin could decrease the excessive growth and specialization of skin abnormalities in a manner that depends on the dosage. Indeed, there have been reports indicating that hesperidin suppresses the growth of tumor cells in a manner that is dependent on both the duration and dosage of treatment (141). According to the findings of another study, it was discovered that hesperidin can hinder the production of PASI, the secretion of pro-inflammatory substances in skin lesions, and the excessive growth and specialization of the outermost layer of the skin (62). It has the potential to serve as a therapeutic treatment for serum metabolic problems and skin lesions, hence alleviating the symptoms of psoriasiform dermatitis (62). Furthermore, it has also impacted the process of aerobic respiration in keratinocytes. It has hindered the phosphorylation of IRS-1 Ser312 and the dephosphorylation of Tyr612 in keratinocytes (62). Additionally, it has restored the functionality of IRS-1 and reduced the excessive production of ERK1/2, thereby inhibiting the proliferation of keratinocytes. All these indications imply that hesperidin has a therapeutic function in the treatment of psoriasis (62).

#### 4.3.14 Hispidulin

Hispidulin, also known as 4′,5,7-trihydroxy-6-methoxyflavone, is a kind of flavone compound that is naturally present in many plants, including *Arrabidaea chica*, Crossostephium chinense, and the genera Artemisia and Salvia (63). Hispidulin has a range of pharmacological properties, including antifungal, antioxidant, anti-inflammatory, anti-allergenic, and anti-mutagenic actions (63). The anti-inflammatory action of HPD has been determined through nuclear magnetic resonance tests, specifically due to its methoxy flavone structure. Sang-Hyun Kim and colleagues conducted a study to assess the efficacy of HPD in treating psoriasis. They used an imiquimod (IMQ)-induced animal model and activated keratinocytes for their investigation. The back skin of mice was topically treated with IMQ for six consecutive days, while HPD was supplied orally to the mice. Oral administration of HPD was found to inhibit psoriatic characteristics, such as increased skin thickness, psoriasis area severity index, transepidermal water loss, and neutrophil infiltration, based on histological observation and immunological investigation. HPD reduced abnormally elevated levels of immunoglobulin G2a, myeloperoxidase, and tumor necrosis factor-α. The mouse model demonstrated that HPD led to a decrease in the numbers of Th1 and Th17 cells in the spleen. Furthermore, HPD suppressed the expression of Th1- and Th17-related cytokines and chemokines, as well as the activation of mitogen-activated protein kinases and nuclear factor-κB, in activated keratinocytes. To sum up, HPD effectively reduces psoriasis-induced skin inflammation both in live organisms and in laboratory settings. Thus, they propose that HPD could serve as a highly effective therapeutic option for managing psoriasis.

### 4.4 Alkaloids

Alkaloids are a group of natural compounds containing mainly basic nitrogen atoms that are produced by various organisms such as bacteria, fungi, plants, and animals. Compounds such as amino acid peptides, proteins, nucleotides, nucleic acids, amines, and antibiotics are not usually called alkaloids. They are classified according to the predominant C-N backbone structure and include many subclasses of alkaloids, namely pyrrole, pyridine, quinoline, isoquinoline, indole, and other alkaloids. Alkaloids have a wide range of pharmacological activities, including antimalarial, antiasthmatic, anticancer, cholinomimetic, vasodilator, antiarrhythmic, analgesic, antibacterial, and antidiabetic activities.

#### 4.4.1 Berberine

Berberine, also known as berberine, is a quaternary ammonium alkaloid isolated from Coptis chinensis, a traditional Chinese medicine, and is the main active ingredient of Coptis chinensis antibacterial. It has yellow needle-like crystals and is bitter in taste. It is widely distributed in the plant kingdom, and berberine is found in approximately 10 genera of 4 families. Berberine is a botanical alkaloid that has been employed in the treatment of gastrointestinal disorders, particularly bacterial diarrhea. BBR has been demonstrated to possess diverse pharmacological properties in clinical trials, such as anti-hyperglycemic, anti-cancer, and anti-depressant effects. Sun et al. demonstrated that berberine can suppress the cyclin-dependent kinase 4 (CDK4)/6-retinoblastoma (RB)-cell division cycle 6 (CDC6) signaling pathway in keratinocytes, resulting in decreased proliferation of keratinocytes (64). The antiproliferation actions of berberine are achieved by suppressing the activity of JAK1, JAK2, and tyrosine kinase 2 (TYK2), which subsequently prevents the activation of STAT3. Ultimately, we have proven that berberine could hinder the development of psoriasis-like skin lesions generated by imiquimod. Additionally, berberine also prevents the increase in CDC6 (a crucial controller of pre-RC assembly and DNA replication in eukaryotic cells) and p-STAT3 levels in mice (64).

#### 4.4.2 Indirubin

Indirubin, a bisindole compound, is the active ingredient extracted from the leaves of Indigo naturalis. Indigo naturalis has been commonly used in traditional Chinese medicine to treat autoimmune diseases and inflammatory diseases (142, 143). Clinical studies have supported the application of indigo naturalis ointment as an effective treatment for psoriasis (144). The study showed that indolescence ameliorated keratinocyte proliferation, reduced the infiltration of CD3+ T cells, IL-17 A-producing γδ T cells, and CD11b + neutrophils, inhibited the mRNA expression of Il1, Il6, Il23, Il17a, and Il22, and increased the protein expression of Jak/Stat pathway-related molecules in the skin lesion (145). Indirubin also reduced the abundance of γδ T cells and CCR6+ γδ T cells (the major IL-17A producers) in the spleen and lymph nodes. In cultured γδ T cells, indirubin inhibited the mRNA expression of Il17a and Ifng and the secretion of IL-17A while suppressing the activation of the Jak3/Stat3 pathways (145). Another study showed that CD274 was the regulator of the indirubin-mediated effect on mouse psoriasis-like skin lesions based on co-expression network analysis, contributing to the alleviation of mouse psoriasis-like skin lesions (146). At the same time, indirubin combined with human umbilical cord mesenchymal stem cells can also reduce psoriatic lesions in BALB/c mice (147).

## 5 Clinical application of natural products

Various remedies used to treat psoriasis were recorded in Chinese medicinal classics, like TaiPingShengHui Formulas and PuJi Formulas, which can be divided into two parts as formulas for topical or for oral use, respectively. Topical remedies involve preparations manufactured into traditional dosage forms, like creams, oils, unguentum, plaster and lotion decoctions, while oral drugs involve those prepared into decoctions, tablets and pulvis, etc. The use of traditional herbal compound prescriptions for psoriasis treatment are under the guideline of “treatment from blood aspect,” Therefore, herbs that are targeted to “blood” and detoxifcation are usually used. A clinical study on 675 cases (148) made a comparison between Yinxieling Ointment, a topical prescription [Mustard gas, Radix Sangusorbae (Diyu), Radix Scutellariae (Huangqin), Cortex Phellodendri (Huangbai)] and Dichlorodiethyl sulfde, and they found comparatively equal efcacy. For oral prescriptions, the clinical efcacy of HuoXueSanYu Decoction (148) [Rhizoma Sparganii(Sanleng), Curcumae Rhizoma (E’zhu), Semen Persicae (Taoren), Flos Carthami (Honghua), Spatholobi Caulis (Jixueteng), Ramulus Euonymi (Guijianyu), Herba Hedyotis (Baihuasheshecao), Salviae Miltiorrhizae Radix and Rhizoma (Danshen), and Pericarpium Citri Reticulatae (Chenpi)] were found to be comparable to acitretin (10 mg, b.i.d.), for the treatment of psoriasis vulgaris. JiaWeiHuangLianJieDu Decoction (149) [Radix Scutellariae (Huangqin), Cortex Phellodendri (Huangbai), Rhizoma Coptidis (Huanglian), etc.] was reported to alleviate the symptoms in 45 of 50 psoriatic subjects.

In most cases, the formula for anti-psoriatic treatment consists of diferent kinds of herbs, which are used as adjuvant therapy or joint with other drug as combined treatment. Anti-psoriatic herb compound prescriptions commonly combine with Narrow Bound Ultraviolet B(NB-UVB) light to achieve higher efficacy. A study (150) analyzed the effect of Tuiyin Decoction combined with NB-UVB, where one group used combined therapy and two control groups applied NB-UVB or Tuiyin Decoction, respectively, and efficiency rates were revealed to 95.3, 67.4 and 72.1%, respectively, indicating that joint treatment has much higher efficacy. Some psoriasis cases are also treated by chemical drugs jointly with Chinese herbal formulas, such as corticosteroids, calcineurin inhibitors and so on. A study (151) demonstrated that calcipotriol betamethasone ointment combined with PSORI-CM01 Decoction [Paeoniae Radix Rubra (Chishao), Rhizoma Curcumae (Erzhu), Sarcandra (Caoshanhu), Radix Glycyrrhiza (Gancao), Fructus Mume (Wumei), Arnebiae Radix (Zicao), and Rhizoma Smilacis Glabrae (Tufuling)] performed better efcacy and a lower relapse rate than combined with an oral placebo. ZaoShiKuShen Decoction (152) [Radix Sophorae Flavescentis (Kushen), Coix chinensis Tod. (Yiyiren), Rhizoma Smilacis Glabrae (Tufuling), Cortex Phellodendri (Huangbai), etc.] was jointly applied with 0.03% Tacrolimus, which showed 14% higher efcacy than that of 0.03% tacrolimus alone in a 100-patient clinical trial.

In addition, drugs approved by CFDA have also been studied, which contained herbal prescriptions and active composites extracted from herbs in various dosage forms, such as tablets, pills, creams, ointments, patches and injections. In the 34 approved drugs, the majority of them were orally administered two or three times per day, while 17 of them were the same extraction prepared in different dosage forms, such as formula Fufangqingdai, which has been prepared into tablets (Approved Number: Z20150034), pills (Approved Number: Z61020964), condensed pills (Approved Number: Z20080269) and capsules (Approved Number: Z20010157). Tere were also two injections, one containing Radix Sophorae Flavescentis (Kushen) (Approved Number: Z14021231), another involving Fructus Psoraleae (Buguzhi) (Approved Number: Z41022361) and six topical agents. However, from 2004 to June 2012, the National Adverse Drug Reaction Monitoring Center has revealed 344 cases that appeared to have adverse reactions in that digestive system, skin and its afliated glands, and the nervous system after being treated with systemic administered prescriptions, and 23 severe cases involved medicinal liver damage and gastrointestinal bleeding. Compared to systemic preparations, those for topical use showed less adverse events including hotness, redness, dryness or itching on the administered skin, which are usually reversible or self-healing.

## 6 Conclusion and discussion

Until now, those suffering from psoriasis have lacked access to a curative treatment for this ailment. Certain preparations hinder the activity of immune factors or suppress the manifestations of psoriasis. Existing therapeutic agents exhibit inherent limitations, such as patient dissatisfaction stemming from pharmacological inefficacy and potential adverse reactions—namely, mood fluctuations, gastrointestinal disturbances, and emesis. The fight against psoriasis is hindered by the absence of a viable and enduring therapeutic strategy. There is a significant demand for the ongoing advancement of novel, secure, and efficacious therapies for psoriasis. In recent decades, extracts from plants and specific phytochemicals from natural resources have garnered significant interest as potential remedies for psoriasis, among the numerous active molecules that have been investigated. Multiple research projects investigating the use of natural sources for psoriasis treatment have identified promising outcomes, particularly in terms of inhibiting cell growth, alleviating itching, and reducing inflammation cytokine levels. The use of natural substances, as opposed to medication, does not result in patient frustration, mood swings, diarrhea, or vomiting, which is a beneficial aspect of their usage. Currently, most studies on the effectiveness of natural substances in treating psoriasis rely on laboratory experiments or animal models. Several studies indicate that natural products may be effective in treating psoriasis due to their capacity to suppress the growth of keratinocytes. Information regarding the effectiveness of natural products in treating psoriasis is available from various sources, including *in vitro* cellular studies, *in vivo* animal tests, and clinical trials. Currently, biologics are the most efficacious medications. However, their cost is prohibitively high compared to chemical medications or traditional Chinese medicine, making them unsuitable for long-term treatment. Exploring novel technologies to decrease production expenses is undeniably a prevailing trend in the development of monoclonal antibody therapeutics. In contrast, when compared to chemical and biological treatments, traditional Chinese medicine and its active extracts are more cost-effective and have fewer negative side effects. This makes them more appropriate for treating milder cases of psoriasis.

Traditional Chinese medicine is safe in the treatment of psoriasis, has a low incidence of adverse reactions, and patients have a high degree of tolerance to TCM treatment. In some clinical trials, oral or topical use may reduce the adverse effects of the combined use of western medicine. In terms of oral Chinese medicine, nausea, vomiting, abdominal pain, and diarrhea are common adverse reactions; the incidence of significantly abnormal laboratory parameters is slightly lower when Chinese medicine is used to treat psoriasis. The adverse reactions of external treatment of traditional Chinese medicine can manifest as skin itching, burning, and tingling, but they are lower than the incidence of adverse reactions of external western medicine in the control group, with better safety. No serious adverse events occurred in other external treatments of traditional Chinese medicine. Therefore, traditional Chinese medicine is a safe treatment for psoriasis, and when combined with conventional treatment and drug dosage, it can ensure efficacy while reducing the occurrence of adverse reactions. However, because there is still a lack of larger samples and mechanistic studies on the treatment of psoriasis with traditional Chinese medicine, further studies on this aspect are needed.

Few therapeutic uses exist, even though natural medications are effective anti-psoriasis in psoriasis animal trials. For preclinical evaluation, modern medical animal experiments are typically the default gold standard. However, the outcomes of animal tests are sometimes extremely unsatisfactory or even contradictory to the outcomes of human clinical application, mostly for the following three reasons: (1) how modifications to the laboratory environment affect research findings; (2) how human and animal illness models differ from one another; and (3) how species’ physiologies and genetic makeup differ from one another (153). However, because the pathogenic mechanisms behind psoriasis are so intricate, there are still certain gaps in our understanding of them, which hinders the advancement of clinical treatment. There is currently a dearth of studies on draggability, despite the relative clarity of natural products’ targets and efficacy. Furthermore, the animals employed in earlier research were young and in good health, which might not accurately reflect the circumstances of actual clinical practice. Consequently, the application of Chinese medicine or its small-molecule extractions for treating psoriasis holds great potential and should be further investigated in the future.

Natural products have obvious advantages in the treatment of psoriasis, especially since the combination of novel drug delivery systems and natural products provide a potentially effective the-rapeutic strategy for psoriasis. However, some problems are remaining. The major factor leading to debate is safety issues. In psoriatic lesions, the drug absorption pathway, skin accumulation, and systemic circulation have been significantly changed compared with normal skin. The delivery capacity, efficacy, and safety of a novel drug delivery system should be considered comprehensively to achieve a better therapeutic effect. Until now, most of the natural product for psoriasis is single natural product, which cannot achieve optimal therapeutic. Hence, when the condition is more complex, such as moderate or severe psoriasis, natural product can exert synergistic effect with other biologic agents, but the joint mechanisms of action need to be carried out systematically. Additionally, the limitations of novel drug delivery systems, including low drug loading, physicochemical properties and stability, encapsulation efficiency and industrial production challenge, have hindered the application in clinical trials. Therefore, further studies should focus more attention on these factors to guide their effective therapy for psoriasis. As for the current treatment of psoriasis with natural products combined with novel drug delivery systems, available data on their clinical effectiveness are limited because of most studies being at the preclinical research stage with a single-animal model. Moreover, the existing treatments are mainly for mild or moderate psoriasis rather than for severe psoriasis. The era of precision medicine sets a higher requirement for the treatment of psoriasis. Perhaps the future management of psoriasis can advance in the direction of targeted therapy and precision medicine by targeting specific cells or genes.

Here, we present some of our insights into the conundrum of how the results of experimental animals can be translated into the clinic. Before new drugs enter clinical human trials, there are three important questions: 1. What does the human body do about drugs? 2. What do drugs do to the human body? 3. What do drugs do about diseases? To answer these three questions (metabolism and exposure of drugs in internal targets and other organs and tissues, side effects and safety to humans, treatment of diseases, and benefits to patients), animal experiments are so far insurmountable experimental means. Professor Kimmelman, a biomedical ethics expert, commented in Nature entitled “Consider drug efficacy before first-in-human trials” (153). He concluded that putting drugs that are likely to be ineffective and unbeneficial into clinical trials should be a matter of medical ethics worth exploring, and he proposed that animal studies provide the experimental means to finally “assess whether experimental treatments are promising enough to be tested in humans. “After understanding these three points, we believe that animal models that more closely mimic clinical pathogenesis should be developed, using animal models similar to human metabolism, such as monkeys, pigs, etc., to deeply explore the pharmacokinetics of drugs and other mechanisms. The most important point is that the adequacy of the study rationale and the rationality of the design should be justified before selecting a similar study.
